# Integrating TNF-α with Established Tumor Markers to Enhance Prognostic Accuracy in Gastric Cancer: A Prospective Observational Study

**DOI:** 10.3390/biomedicines13040928

**Published:** 2025-04-09

**Authors:** Mihai Catalin Rosu, Cristi Tarta, Silviu Moldovan, Andreea-Adriana Neamtu, Andrei Ardelean, Marco Capitanio, Diana Herczeg, Ionut-Flaviu Faur, Renata Bende, Luminita Pilat, Virgiliu Mihai Prunoiu, Carmen Neamtu, Bogdan Dan Totolici

**Affiliations:** 1 Surgery Department, Clinical County Emergency Hospital of Arad, Andrenyi Karoly Str., No. 2-4, 310037 Arad, Romania; mihai.roshu@yahoo.com (M.C.R.); andreea.neamtu@umft.ro (A.-A.N.); andreiardelean86@gmail.com (A.A.); dianaherczeg09@gmail.com (D.H.); neamtu.carmen@uvvg.ro (C.N.); totolici_bogdan@yahoo.com (B.D.T.); 2 Faculty of Medicine, “Vasile Goldis” Western University of Arad, Liviu Rebreanu Str., Nr. 86, 310045 Arad, Romania; silviu602@gmail.com (S.M.); luminita.pilat@yahoo.com (L.P.); 3 Department X, Discipline of General Surgery II, Victor Babes University of Medicine and Pharmacy Timisoara, E. Murgu Square, No. 2, 300041 Timisoara, Romania; capitanio.marco@umft.ro (M.C.); flaviu.faur@umft.ro (I.-F.F.); 4 Department of Toxicology, “Victor Babes” University of Medicine and Pharmacy, E. Murgu Square, No. 2, 300041 Timisoara, Romania; 5 Research Centre for Pharmaco-Toxicological Evaluation, “Victor Babes” University of Medicine and Pharmacy, E. Murgu Square, No. 2, 300041 Timisoara, Romania; 6 Center for Advanced Research in Gastroenterology and Hepatology, Department of Internal Medicine II, Division of Gastroenterology and Hepatology, “Victor Babes” University of Medicine and Pharmacy Timisoara, E. Murgu Square, No. 2, 300041 Timisoara, Romania; renatabende29@gmail.com; 7 Clinical Department No. 10, General Surgery, University of Medicine and Pharmacy “Carol Davila”, 050474 Bucharest, Romania; virgiliuprunoiu@yahoo.com; 8 Department of Oncological Surgery, Oncological Institute “Prof. Dr. Alexandru Trestioreanu”, 022328 Bucharest, Romania

**Keywords:** gastric cancer biomarkers, diagnosis and treatment, gastric cancer metastasis, tumor microenvironment, carcinoembryonic antigen (CEA), cancer antigen 19-9 (CA19-9), cancer antigen 72-4 (CA72-4), alpha-fetoprotein (AFP), tumor necrosis factor alpha (TNF-α), inflammation and inflammatory parameters

## Abstract

**Background/Objectives**: Gastric cancer remains a leading cause of cancer mortality worldwide. Reliable biomarkers are crucial for early detection, prognostication, and therapy monitoring. While classical tumor markers such as carcinoembryonic antigen (CEA), cancer antigen (CA)19-9, CA72-4, and alpha-fetoprotein (AFP) are used in clinical practice, their accuracy can be limited. Tumor necrosis factor alpha (TNF-α) is an inflammatory cytokine implicated in tumor progression, yet its relationship with established gastric cancer tumor markers has not been fully clarified. This study aimed to determine whether elevated TNF-α correlates with key tumor markers and disease stage in gastric cancer. **Methods**: In this prospective observational study, we enrolled 80 gastric cancer patients and 20 non-neoplastic controls. Baseline clinical data, laboratory parameters, and tumor markers (CEA, CA19-9, CA72-4, AFP) were recorded. TNF-α concentrations were measured using enzyme-linked immunosorbent assays. Correlation analyses and multivariate regression were performed to assess the relationship of TNF-α with tumor markers, inflammatory indices, and disease stage. **Results**: TNF-α was significantly elevated in gastric cancer patients (median 4.5 pg/mL) compared to controls (2.9 pg/mL). TNF-α showed a robust correlation with CA19-9 (rho = 0.502) and CA72-4 (rho = 0.385), and a moderate correlation with CEA (rho = 0.279). TNF-α concentrations were highest in Stage IV disease and in the intestinal-type histology. In regression analysis, only CA19-9 and CA72-4 remained independent predictors of TNF-α after controlling for clinical confounders. **Conclusions**: TNF-α is strongly associated with CA19-9 and CA72-4 and rises with advancing stage, highlighting its potential as an adjunct marker for assessing gastric cancer burden. These findings provide a rationale for further research on TNF-α as both a prognostic biomarker and a possible therapeutic target in gastric cancer.

## 1. Introduction

Gastric cancer remains a significant global health challenge, with high mortality rates despite advances in treatment modalities. Early detection and accurate prognostication are crucial for optimizing treatment strategies; i.e., carefully balancing between treatment efficacy and toxicity [1]. While conventional tumor markers such as carcinoembryonic antigen (CEA), cancer antigen (CA)19-9, CA72-4, and alpha-fetoprotein (AFP) are widely used in clinical practice, their utility is limited by moderate sensitivity and specificity. This has driven the search for novel biomarkers that could enhance prognostic accuracy and provide insights into disease biology [2].

Globally, gastric cancer is diagnosed in more than 1 million people annually, with approximately 770,000 deaths in 2020 [3]. It ranks among the top causes of cancer mortality. Major risk factors include chronic Helicobacter pylori infection—a Group I carcinogen linked to ~90% of distal gastric cancers—as well as high-salt diets, smoking, and obesity. These factors contribute to gastric carcinogenesis and regional incidence variations [3].

Tumor necrosis factor alpha (TNF-α) represents a key mediator in inflammatory [4,5], infectious [6,7,8], and carcinogenic processes [9,10,11]. This pro-inflammatory cytokine is produced by a diverse array of cells, including macrophages, monocytes, lymphocytes, mast cells, neutrophils, keratinocytes, and smooth muscle cells. TNF-α release is triggered when macrophages, CD4+ T cells, and natural killer (NK) cells encounter lipopolysaccharides, bacterial products, and interleukin 1 [12]. In gastric cancer, TNF-α exhibits a dual nature: it can promote tumor progression through angiogenesis, cellular proliferation, and infiltration, while also possessing the capacity to induce apoptosis under specific conditions [13]. Moreover, previous studies have identified TNF-α as a potential predictive factor for response to therapy and overall disease progression during oncological treatment, specifically through modulation of the immunological response in the tumor microenvironment [6,7,8]. Recent research has identified TNF-α polymorphisms, particularly TNF-α 308, 857, and 238, as potential risk factors for gastric cancer, with notable significance in East Asian populations [14]. These genetic variants, especially when interacting with environmental factors like smoking, have been extensively studied in relation to gastric cancer risk [15].

CEA, a glycoprotein comprising approximately 40% protein and 60% carbohydrates with a molecular weight of 200 kDa, represents one of the most widely studied oncofetal antigens. During embryonic and fetal development, CEA is produced in high concentrations in the gastrointestinal tract and pancreas. In adults, while low concentrations can be detected in normal intestinal mucosae, exocrine pancreas, and liver tissue, significantly elevated levels are observed in colorectal and gastric adenocarcinomas [16]. A comprehensive meta-analysis by Deng et al. demonstrated that elevated pretreatment serum CEA levels correlate strongly with poor prognosis in gastric cancer patients, effectively doubling mortality risk [17].

CA72-4, a tumor-associated glycoprotein complex, with elevated expression in various types of neoplasms, has demonstrated particular relevance in gastric cancer diagnostics and monitoring. Its high-molecular-weight mucin-like antigen (TAG-72) is detected using specific monoclonal antibodies and has shown superior specificity for gastric cancer compared to other markers. Clinical studies have consistently demonstrated that elevated CA72-4 levels correlate with advanced disease stages and poorer survival outcomes [18].

CA19-9, identified by the monoclonal antibody 1116-NS-19-9, though initially recognized for its utility in pancreatic and biliary tract cancers, has also proven valuable in gastric cancer management. Elevated CA19-9 levels in gastric cancer patients correlate significantly with advanced disease stages and distant tumor spread, manifesting as either lymph node or organ metastases. Preoperative elevation of CA19-9 serum levels serves as an independent indicator of poor prognosis and lower survival rates [19].

AFP is a glycoprotein primarily produced by the fetal liver and yolk sac during embryonic development. While AFP is widely recognized as a tumor marker for hepatocellular carcinoma and germ cell tumors, emerging evidence suggests its relevance in gastric cancer, particularly in a rare subtype known as AFP-producing gastric cancer (AFPGC). This variant, accounting for approximately 1.3–15% of all gastric cancers, is characterized by aggressive behavior, including high rates of vascular invasion, lymph node metastasis, and liver metastasis [20,21]. Elevated AFP levels in gastric cancer patients have been associated with poor differentiation, advanced disease stage, and shortened overall survival [20,21]. Additionally, AFPGC exhibits distinct pathological features and may respond differently to chemotherapy compared to conventional gastric adenocarcinomas [20]. However, the role of AFP as a general prognostic marker in gastric cancer beyond AFPGC remains contentious, with some studies indicating potential utility in disease monitoring, while others highlight its limited sensitivity and specificity [20,21].

The combined evaluation of these markers offers enhanced diagnostic and prognostic value compared to individual marker assessment [22]. This cumulative approach proves particularly valuable in follow-up settings, where tracking marker fluctuations from baseline can facilitate early detection of metastasis [23]. The prognostic utility of these markers extends beyond gastric cancer, demonstrating value in estimating overall survival and detecting recurrence across various types of malignancies [24,25].

Recent studies have begun exploring the relationship between TNF-α and these established tumor markers, with preliminary evidence suggesting that elevated TNF-α levels correlate with worse overall and 5-year survival rates [26,27]. While previous studies by the authors have examined various aspects of tumor marker interactions in gastric cancer, the specific relationship between TNF-α and the combined serological panel of AFP, CEA, CA19-9, and CA72-4 remains unexplored [2,28].

The present study aimed to investigate the correlations between plasma TNF-α levels and established tumor markers (AFP, CEA, CA72-4, and CA19-9) in gastric cancer patients, with particular attention to how these relationships vary across disease stages. Understanding these interactions could enhance prognostic accuracy and potentially guide therapeutic decision-making, particularly regarding anti-inflammatory interventions. This is supported by evidence from anti-TNF therapies in other malignancies, which have shown promising results in reducing tumor progression [29,30]. Additionally, this re-search seeks to elucidate how these markers relate to various clinical, pathological, and biochemical parameters, potentially offering new insights into disease progression mechanisms and treatment response prediction.

## 2. Materials and Methods

### 2.1. Study Design and Population

This prospective observational study was conducted at the Arad County Emergency Clinical Hospital over a 4-year period, from January 2020 to January 2024. All study procedures were approved by the Research Ethics Committee of the Vasile Goldiș Western University of Arad (13/18.03.2020). The study followed the STROBE guidelines for observational research.

We enrolled consecutive adult patients (≥18 years old), with a clinical or histopathological diagnosis of gastric malignancy, admitted within our institution’s General Surgery Department, undergoing either surgical or conservative treatment. Exclusion criteria consisted of (a) coexisting autoimmune or chronic inflammatory disorders likely to elevate tumor marker or TNF-α levels, (b) gastrointestinal stromal tumors (GIST), and (c) other active malignancies or incomplete data regarding diagnosis or treatment status. The control group consisted of age-matched patients admitted for non-neoplastic surgical conditions during the same timeframe. All participants provided written informed consent for TNF-α assessment and use of all other relevant clinical data.

Sample size was calculated based on the primary outcome measure (correlation between TNF-α and tumor markers), using G*Power (version 3.1.9.7, Heinrich Heine University, Düsseldorf, Germany). Assuming a moderate correlation (r ≈ 0.3) between TNF-α and the selected tumor markers, at α = 0.05 with 80% power, yielded a minimum of 80 patients for the gastric cancer arm, which was achieved after applying inclusion/exclusion criteria. The control group size was set at 20 subjects to achieve a 4:1 case-control ratio.

### 2.2. Data Collection and Study Variables

Two trained researchers independently extracted data from electronic medical records using a standardized form. Data included demographic information, clinical characteristics, laboratory results, imaging findings, surgical procedures, and pathology reports. Discrepancies were resolved through consensus with a third reviewer.

#### 2.2.1. Clinical Characteristics

All patients underwent standardized clinical evaluation including medical history, physical examination, and performance status assessment. Disease staging followed the 8th edition of the AJCC TNM classification system. Radiological staging included contrast-enhanced computed tomography of the chest, abdomen, and pelvis. All available imaging studies were reviewed, with particular attention to CT findings detailing tumor characteristics, local invasion, lymph node involvement, and distant metastases. Each patient underwent upper gastrointestinal endoscopy (esophagogastroduodenoscopy, EGD) for direct visualization of gastric lesions and histopathological biopsy confirmation. Additional imaging studies, such as PET-CT or MRI, were documented when available. This combined diagnostic strategy enabled accurate classification of disease stage, reflecting current best-practice guidelines from the European Society for Medical Oncology (ESMO).

#### 2.2.2. Surgical Interventions

Patients deemed suitable for curative or palliative surgery underwent open or laparoscopic procedures based on tumor location and extent. Distal subtotal gastrectomy, typically involving two-thirds stomach resection, was performed for distal tumors; total gastrectomy was applied when lesions extended beyond the mid-stomach or involved the cardia. The extent of lymph node dissection (D1, D1+, or D2) was determined according to each patient’s intraoperative and imaging findings, balancing oncologic completeness with surgical safety. Reconstruction methods included Billroth I or Billroth II gastrojejunostomy, and Roux-en-Y anastomosis in total gastrectomy cases. If curative resection was infeasible (e.g., advanced obstructive disease, peritoneal carcinomatosis), palliative procedures such as gastrojejunostomy or feeding gastrostomy/jejunostomy were undertaken, to alleviate symptoms and support nutrition. Splenectomy was added when necessary to achieve negative resection margins or address splenic hilum infiltration.

#### 2.2.3. Pathology Assessment

Resected or biopsy specimens were processed at the institution’s pathology laboratory. Histopathological parameters included tumor subtype classified by World Health Organization (WHO) criteria (tubular adenocarcinoma, poorly cohesive carcinoma, carcinoma NOS, adenocarcinoma NOS, and other variants) and Lauren classification (intestinal, diffuse, or mixed/undifferentiated), tumor grade (G1–G3), depth of invasion (pT), nodal status (pN), and the presence of lymphovascular (LVI) or perineural (PNI) invasion. Margins were designated as R0 (negative), R1 (microscopic residual disease), or R2 (macroscopic residual disease). In patients receiving neoadjuvant treatment, the final pathologic stage accounted for any therapy-induced changes. Nodal dissection was assessed according to standard guidelines, and the classification encompassed both staging (cTNM) and pathological staging (pTNM) where applicable.

#### 2.2.4. Standard Bloodwork

At admission or preoperatively, peripheral blood samples were collected from each participant under fasting conditions. For standard laboratory analyses, samples were processed within 1 h of collection in the RENAR-accredited laboratory of Arad County Emergency Clinical Hospital. These included complete blood count, blood group typing (ABO and Rh), liver and kidney function tests (aspartate aminotransferase (AST), alanine aminotransferase (ALT), gamma-glutamyl transferase (GGT), alkaline phosphatase (ALP), bilirubin, urea, creatinine), procalcitonin, inflammatory markers (C-reactive protein (CRP), erythrocyte sedimentation rate (ESR), fibrinogen), and total plasma proteins.

Hematological analysis was performed using the Sysmex XN 1500 analyzer (Sysmex Corporation, Kobe, Japan), which employs fluorescence flow cytometry technology. Complete blood count parameters included hemoglobin, hematocrit, red blood cell count, white blood cell count with 5-part differential, platelet count, and red cell indices. The analyzer underwent daily quality control using XN Check (Sysmex Corporation, Milton Keynes, UK) at three levels (low, normal, high). Biochemical parameters were analyzed on the Cobas 6000 c501 module (Roche Diagnostics, Mannheim, Germany). Inflammatory markers were measured using the immunoturbidimetric method on a Cobas 6000 c501 for CRP; the Westergreen method (VES-Matic Cube 30, DIESSE Diagnostica Senese, Siena, Italy) for ESR; and the electrochemiluminescent immunoassay (ECLIA) method on a Cobas e601 module for procalcitonin.

#### 2.2.5. Tumor Biomarkers

Established tumor markers (CEA, CA19-9, CA72-4, AFP) were processed within the aforementioned clinical laboratory of Arad County Emergency Clinical Hospital, while TNF-α was assessed from matched samples at the biochemistry laboratory of the Vasile Goldiș Western University of Arad. All biomarker analyses were performed on blood samples collected in serum separator tubes before any therapeutic intervention. Samples were allowed to clot for 30 min at room temperature and centrifuged at 3000× *g* for 15 min. Serum was analyzed immediately or stored at −20 °C for no longer than 2 months.

The following markers were analyzed on the Cobas e601 analyzer (Roche Diagnostics, Mannheim, Germany), using ECLIA technology:

CEA: detection range 0.2–1000 ng/mL, reference range <5.0 ng/mL;

CA19-9: detection range 0.6–1000 U/mL, reference range <37 U/mL;

AFP: detection range: 0.5–1000 ng/mL, reference range <7.0 ng/mL.

The Cobas e601 system utilized ruthenium-based electrochemiluminescent detection technology with streptavidin-coated microparticles. Two-point calibration and quality control were performed every 24 h using PreciControl Tumor Marker (Roche Diagnostics).

CA72-4 was analyzed on the Sunrise™ microplate reader (Tecan Trading AG, Männedorf, Switzerland), using commercially available enzyme-linked immunosorbent assay (ELISA) kits (Fujirebio Diagnostics, Malvern, PA, USA), with a detection range of 1–100 U/mL and reference range <6.9 U/mL. On a similar platform, TNF-α was measured, also using commercial ELISA kits (R&D Systems, Minneapolis, MN, USA), with a detection range of 0.5–100 pg/mL and reference range <3.8 pg/mL. For ELISA assays, each patient sample was tested in duplicate and the average value was reported to ensure accuracy. Furthermore, each run included calibrators and controls provided by the manufacturers. Standard curves were generated using a 4-parameter logistic curve fit. Samples above the analytical measurement range were diluted and retested according to manufacturer protocols.

All tumor marker analyses included daily internal quality control at two levels (normal and pathological range). External quality assessment was performed through participation in the RENAR proficiency testing program, with acceptable performance documented throughout the study period. Results were considered valid only when quality control criteria were met and samples showed no signs of hemolysis, lipemia, or other interfering conditions.

### 2.3. Statistical Analysis

All statistical analyses were conducted using MedCalc (version 19.4, MedCalc Software Ltd., Ostend, Belgium) and Microsoft Excel 2019. Normality of continuous variables was determined by the Kolmogorov–Smirnov test. Normally distributed variables were reported as mean ± standard deviation (SD) and compared using Student’s *t*-tests. Non-normally distributed data were represented as medians and interquartile ranges (IQR) and tested with the Mann–Whitney U or Kruskal–Wallis tests (for multiple-group comparisons). The chi-square test or Fisher’s exact test was used for categorical variables.

Correlation analyses between TNF-α and other tumor markers (CEA, CA19-9, CA72-4, AFP) or laboratory parameters (CRP, ESR, fibrinogen, hemoglobin, total proteins) used Spearman’s rank test. Multiple comparisons were controlled by the Benjamini–Hochberg procedure, with a false discovery rate set at 5%. In specific cases (e.g., high number of comparisons), Bonferroni adjustment was performed. Significance was set at *p* < 0.05 (two-tailed).

## 3. Results

### 3.1. Comparative Patient Characteristics and Laboratory Findings

The study included 80 patients with gastric cancer and 20 matched controls, as seen in Table 1. In the cancer group, the median age was 68 years [IQR: 60–76], with 49 males (61.3%) and 31 females (38.7%). The matched control group had comparable demographics with a median age of 66.5 years [61.25–68.75] and equal gender distribution (50% male, 50% female). Regarding surgical management, 63 patients (78.8%) from within the study group underwent proper surgical procedures. Of these, 45 patients (56.3%) had curative intent surgery including total or subtotal gastrectomy, while 18 patients (22.5%) underwent palliative procedures (gastroenterostomy, feeding tube placement, or exploratory surgery). The remaining 17 patients (21.2%) underwent biopsies or did not undergo surgical interventions in our center; i.e., were managed conservatively for disease complications.

According to WHO classification, tubular adenocarcinoma was the most frequent histological type (30 cases, 37.5%), followed by adenocarcinoma not otherwise specified (NOS) (24 cases, 30.0%), carcinoma NOS (15 cases, 18.8%), poorly cohesive carcinoma with signet ring cell type (5 cases, 6.3%), and other variants (6 cases, 7.5%, including pleomorphic carcinoma, mixed neuroendocrine–non-neuroendocrine neoplasm, squamous cell carcinoma, mucinous adenocarcinoma, adenocarcinoma with mixed subtypes). Using Lauren’s classification, the diffuse type was predominant with 33 cases (41.3%), followed by intestinal type with 31 cases (38.8%), and mixed/undifferentiated type with 16 cases (20.0%). Regarding tumor differentiation, 42 cases (52.5%) were G3, 33 cases (41.3%) were G2, and 5 cases (6.3%) were G1. 

Disease staging according to the AJCC 8th edition revealed Stage IV in 34 patients (42.5%), Stage III (A/B/C) in 24 patients (30.0%), Stage II (A/B) in 15 patients (18.8%), and Stage I (A/B) in 7 patients (8.8%). The TNM classification showed T4a/b in 44 cases (55.0%), T2 in 22 cases (27.5%), T3 in 11 cases (13.8%), and T1a in 3 cases (3.8%). Nodal involvement showed N1 in 29 cases (36.3%), N2 in 22 cases (27.5%), N0 in 15 cases (18.8%), N3a/b in 13 cases (16.3%), and Nx in 1 case (1.3%). Distant metastases (M1) were present in 34 cases (42.5%). 

As seen in Table 1, patients with gastric cancer showed notable alterations in baseline laboratory parameters compared to controls. Hemoglobin levels were significantly lower in the cancer group (10.5 [9.2–12.2] vs. 13.9 [13.2–14.9] g/dL, *p* < 0.001), as were total serum proteins (6.1 [5.4–6.7] vs. 6.9 [6.6–7.4] g/dL, *p* < 0.001) and albumin (3.4 [2.9–3.9] vs. 4.2 [3.8–4.5] g/dL, *p* < 0.001). Liver function tests (ALT, AST, GGT, ALP) and renal function parameters (creatinine) showed no significant differences between groups, suggesting preserved organ function in most patients at the time of diagnosis.

Among inflammatory markers, CRP (16.2 [5.6–44.0] vs. 1.6 [1.2–4.2] mg/L, *p* < 0.001), ESR (26.0 [18.0–42.0] vs. 15.0 [10.0–25.0] mm/h, *p* < 0.001), and fibrinogen (419.0 [350.0–509.0] vs. 329.0 [286.0–398.0] mg/dL, *p* = 0.003) were all significantly elevated in the cancer group. Procalcitonin levels were also significantly higher in cancer patients (0.06 [0.04–0.10] vs. 0.03 [0.02–0.04] ng/mL, *p* < 0.001).

Regarding tumor markers, CA72-4 showed the most significant elevation (4.9 [3.1–7.9] vs. 2.4 [1.8–4.2] U/mL, *p* < 0.001), followed by CA19-9 (12.1 [6.1–25.1] vs. 6.4 [3.2–10.6] U/mL, *p* = 0.030) and TNF-α (4.5 [2.8–11.4] vs. 2.9 [1.7–5.7] pg/mL, *p* = 0.014). CEA levels were higher in the cancer group but did not reach statistical significance (2.5 [1.5–5.5] vs. 2.0 [1.3–2.9] ng/mL, *p* = 0.077). Similarly, AFP showed no significant difference between groups (1.7 [1.2–3.1] vs. 1.5 [1.0–2.4] ng/mL, *p* = 0.126).

### 3.2. Overall TNF-α Correlation Analysis

#### 3.2.1. Study Group Correlations

**Figure 1:**
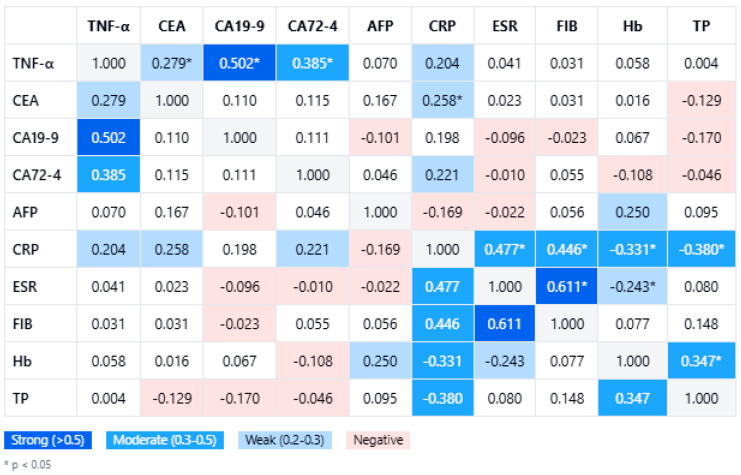
Study group correlation matrix of the studied variables (TNF-α, tumor markers, inflammatory parameters, hemoglobin, and total proteins). Abbreviations: CRP = C-reactive protein; ESR = erythrocyte sedimentation rate; FIB = fibrinogen; Hb = hemoglobin; TP = total proteins; TNF-α = tumor necrosis factor alpha; CEA = carcinoembryonic antigen; CA = cancer antigen; AFP = alpha-fetoprotein.

Within the gastric cancer cohort (n = 80), TNF-α levels demonstrated significant positive correlations with several tumor markers. In Figure 1, a comprehensive correlation matrix further highlights the significant pairwise associations, with TNF-α clustering alongside the tumor burden markers. The strongest association was observed with CA19-9 (Spearman’s rho = 0.502, *p* < 0.001), followed by CA72-4 (rho = 0.385, *p* < 0.001) and CEA (rho = 0.279, *p* = 0.012). In contrast, no meaningful correlation was found between TNF-α and AFP (rho = 0.070, *p* = 0.482). These relationships remained statistically significant after correcting for multiple comparisons using the Benjamini–Hochberg procedure (false discovery rate 5%). Notably, the key correlations with CA19-9 and CA72-4 persisted even under a stringent Bonferroni adjustment (adjusted α ≈ 0.00625), underscoring the robustness of these associations.

In Figure 1, TNF-α levels also show links with systemic inflammatory markers, although these were generally weaker. Among inflammation indices, CRP had the highest correlation with TNF-α (rho = 0.204), but this did not reach significance (*p* = 0.069). Neither ESR (rho = 0.049, *p* = 0.666) nor fibrinogen (rho = 0.030, *p* = 0.792) demonstrated any significant correlation with TNF-α. This suggests that gastric cancer-related TNF-α elevations may be driven more by localized tumor biology than by systemic acute-phase responses. From a clinical perspective, these findings reinforce TNF-α as a potentially valuable biomarker in tandem with CA19-9 and CA72-4, particularly in monitoring or characterizing more advanced gastric malignancies.

#### 3.2.2. Comparative Analysis

When comparing the gastric cancer cohort (n = 80) with the control group (n = 20), clear distinctions emerged in the correlation patterns between TNF-α and key clinical variables (Table 2). In the cancer cohort, TNF-α significantly correlated with several tumor markers—particularly CA19-9 and CA72-4—underscoring TNF-α’s role in tumor-related processes. Conversely, in the control population, none of these markers reached statistical significance in relation to TNF-α. Although CA19-9 displayed a moderate, positive correlation (rho = 0.370, *p* = 0.1084) in controls, it fell short of significance, suggesting that the elevated association in the cancer group was primarily driven by malignancy-associated biology rather than baseline population variation. Thus, Table 2 demonstrates that the significant correlations between TNF-α and markers (CEA, CA19-9, CA72-4) appeared only in the gastric cancer group and not in controls, underscoring that these associations were cancer-specific.

Similar discrepancies were seen for CEA, which demonstrated a moderate positive correlation in gastric cancer patients (rho = 0.279, *p* = 0.0122), but essentially no relationship in controls (rho = −0.020, *p* = 0.9348). Interestingly, while AFP failed to correlate with TNF-α among cancer patients, it approached a moderate correlation (rho = 0.383, *p* = 0.0956) in controls, though still not significant. Collectively, these findings point to the specificity of TNF-α–tumor marker associations in active malignancy.

With respect to inflammatory markers (CRP, fibrinogen) and nutritional parameters (hemoglobin, total proteins), no significant TNF-α correlations were identified in either group. In healthy subjects, this indicates that low baseline TNF-α levels are neither driven by acute-phase reactants nor robustly linked with routine nutritional indices. Taken together, these results reinforce the notion that TNF-α is preferentially tied to tumor-specific factors, whereas its variation in individuals without gastric cancer remains minimal and clinically negligible.

**Figure 2:**
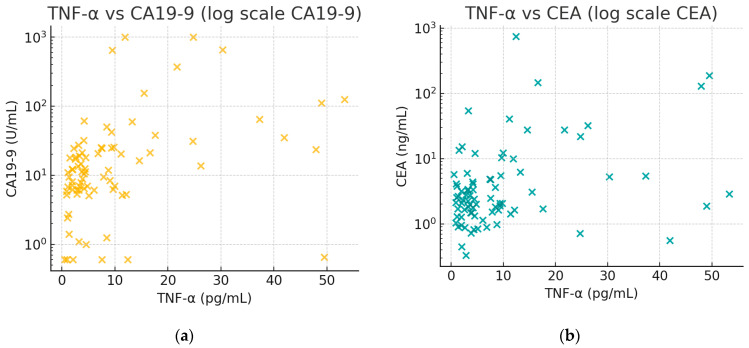
Joint distributions of TNF-α with tumor markers: (**a**) TNF-α (x-axis, pg/mL) vs. CA19-9 (y-axis, U/mL); (**b**) TNF-α (x-axis, pg/mL) vs. CEA (y-axis, ng/mL). Each panel shows a scatterplot (log-scale y-axis) of individual patient values; both TNF-α and the tumor markers tend to increase together, consistent with the correlations noted.

Figure 2 illustrates the positive associations between TNF-α and tumor markers. In Figure 2a, there is an observable trend that patients with higher CA19-9 tend to have higher TNF-α. In Figure 2b, a similar trend for CEA can be seen, though less pronounced than with CA19-9, aligning with the moderate correlation (rho ≈ 0.28).

### 3.3. TNF-α Patterns Across Patient Subgroups

#### 3.3.1. Histological Subtypes

**Figure 3:**
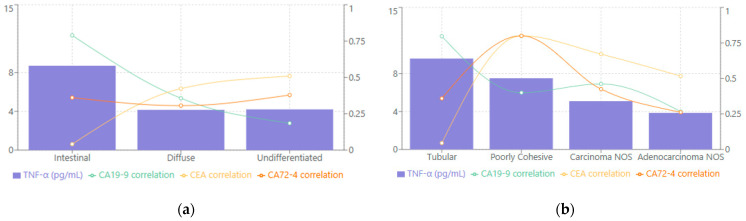
TNF-α median levels and tumor marker correlations stratified by (**a**) Lauren classification; (**b**) WHO classification.

When evaluated according to Lauren classification, TNF-α levels varied substantially among the different subtypes (see Figure 3a). The intestinal variant demonstrated the highest median levels at 8.70 [2.10–21.70] pg/mL, while the undifferentiated and diffuse types showed comparatively lower values, with medians of 4.20 [1.92–9.30] pg/mL and 4.15 [3.12–9.00] pg/mL, respectively. Correlation analyses revealed that the intestinal subtype displayed the strongest relationship between TNF-α and CA19-9 (rho = 0.789, *p* < 0.001), suggesting that this particular histological pattern may be especially prone to heightened proinflammatory cytokine activity in conjunction with elevated tumor markers. The diffuse subtype demonstrated a moderate correlation between TNF-α and CEA (rho = 0.423, *p* < 0.001), while the undifferentiated subtype exhibited a strong correlation with CEA (rho = 0.509, *p* < 0.001). These findings underscore that each Lauren category features a distinct TNF-α expression pattern that can reflect different biological underpinnings of tumor progression.

As seen in Figure 3b, when the WHO classification was applied, tubular adenocarcinoma had the highest median TNF-α at 9.60 [3.02–21.70] pg/mL, followed by poorly cohesive carcinoma at 7.52 [3.53–12.39] pg/mL, carcinoma NOS at 5.10 [3.56–9.30] pg/mL, and adenocarcinoma NOS at 3.86 [1.92–9.30] pg/mL. Correlation patterns further emphasized subtype-specific distinctions. TNF-α in tubular adenocarcinoma showed a particularly robust association with CA19-9 (rho = 0.797, *p* < 0.001), indicating that these tumors may rely more heavily on inflammatory mediators alongside tumor marker elevation. Poorly cohesive carcinoma, on the other hand, demonstrated strong correlations between TNF-α and both CEA and CA72-4 (rho = 0.800, *p* < 0.001 for both), suggesting that different molecular pathways might drive the immunologic and tumor-marker interplay in these more aggressive forms of gastric carcinoma.

#### 3.3.2. Disease Stage-Specific Analysis

The distribution of tumor markers and TNF-α levels across different disease stages revealed several noteworthy trends, as summarized in Table 3. TNF-α exhibited a significant stage-dependent pattern (*p* = 0.037), with the highest median level in Stage IV disease (7.99 pg/mL). Stages II and III demonstrated comparable median TNF-α values (4.17 and 4.14 pg/mL, respectively), suggesting that cytokine elevation may temporarily plateau prior to a marked rise in advanced disease. When broken down by individual T, N, and M categories, median TNF-α values were highest in T4 and M1 lesions, suggesting that extensive local invasion and metastatic disease are associated with elevated TNF-α. For nodal status, N3 disease generally showed higher TNF-α than N0–N2, although the differences did not always reach significance after multiple testing correction. CEA also varied significantly by stage (*p* = 0.007), showing a pronounced increase in Stage IV (median 3.71 ng/mL) relative to Stage III (1.67 ng/mL) and substantial interpatient variability among those with extensive disease (IQR 1.63–21.93 ng/mL).

Although CA19-9 displayed broad changes across stages, these did not reach statistical significance (*p* = 0.084). The particularly wide interquartile range observed in Stage I (n = 7) likely reflects the small patient sample in that category rather than true biological variation. By contrast, CA72-4 and AFP remained relatively stable throughout Stages I to IV (*p* = 0.330 and *p* = 0.195, respectively), indicating limited usefulness for determining disease stage. CRP emerged as another parameter of note (*p* = 0.044), with higher median values observed in Stage IV, pointing to an upsurge in systemic inflammation in late-stage disease. ESR and fibrinogen showed no significant changes, suggesting that they may not reflect or differentiate stage progression in this cohort.

**Figure 4:**
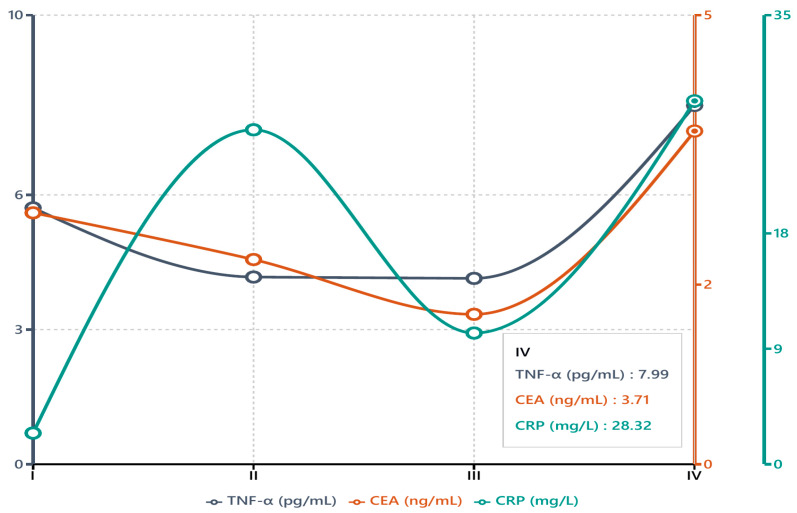
Median TNF-α, CEA, and CRP levels stratified by disease stage.

Figure 4 depicts the median values of TNF-α, CEA, and CRP, plotted across Stages I–IV to highlight the differences identified in Table 3. Notably, TNF-α and CEA levels rose sharply by Stage IV, both roughly doubling from earlier-stage levels, whereas CRP exhibited a milder increase that likewise peaked in advanced disease. This divergence suggests that TNF-α (like CEA) is closely associated with tumor burden in advanced disease, while CRP—an acute-phase reactant—increases with inflammation more generally and less dramatically with stage. However, it is important to recognize that the distribution of patients across stages was not uniform; Stage IV comprised 34 cases, whereas only 7 patients had Stage I disease. This imbalance can limit the statistical power for early-stage comparisons and should be considered when interpreting the stage-specific data.

### 3.4. Multiple Regression Analysis

To further delineate the independent relationships between TNF-α and the variables of interest, a multivariate regression analysis was performed with TNF-α as the outcome and all key markers and staging variables entered as predictors. The model included the four tumor markers (CEA, CA19-9, CA72-4, AFP), the inflammatory markers (CRP, ESR, fibrinogen), and the AJCC stage (treated as an ordinal variable from I to IV). Together, these predictors explained a significant proportion of the variance in TNF-α levels (overall model F {8,71} = 5.06, *p* < 0.001). The model R^2^ was 0.36 (adjusted R^2^ = 0.30), indicating that roughly one-third of the variability in TNF-α concentrations in gastric cancer patients could be accounted for by this set of clinical and laboratory factors. Diagnostics confirmed that regression assumptions were adequately met: residuals showed no marked deviations from normality or homoscedasticity, and no high multicollinearity was detected (all variance inflation factor values < 2.0).

Table 4 presents the regression coefficients, 95% confidence intervals, and significance levels for each predictor. CA19-9 and CA72-4 emerged as the only independent significant predictors of TNF-α levels in the multivariable model. Specifically, for each 1 U/mL increase in CA19-9, the model predicted an increase of approximately 0.10 pg/mL in TNF-α (β = 0.10, 95% CI 0.05–0.15, *p* < 0.001). Similarly, each 1 U/mL increase in CA72-4 was associated with a 0.50 pg/mL rise in TNF-α (β = 0.50, 95% CI 0.18–0.82, *p* = 0.003). These effects remained statistically significant after adjusting for all other variables in the model. In contrast, CEA did not retain a significant independent effect on TNF-α (β = 0.30, 95% CI–0.03–0.63, *p* = 0.075) once the contributions of CA19-9 and CA72-4 were accounted for. This suggests that the observed univariate correlation between TNF-α and CEA was largely mediated by their mutual association with the other tumor markers (particularly CA19-9 and CA72-4). Likewise, AFP showed no independent relationship with TNF-α in the multivariate context (β = 0.01, 95% CI–0.05–0.07, *p* = 0.70).

Thus, as shown in Table 4, only CA19-9 and CA72-4 emerged as independent predictors of TNF-α levels in multivariate analysis (both *p* < 0.01), while other factors (CEA, CRP, stage, etc.) did not retain significance. This indicates that CA19-9 and CA72-4 had the dominant influence on TNF-α among the variables tested.

None of the inflammatory markers or staging variable demonstrated a significant independent association with TNF-α after controlling for the tumor markers. CRP, ESR, and fibrinogen all had small regression coefficients (each β close to 0, *p* > 0.5), indicating that systemic inflammation measures did not contribute additional explanatory power for TNF-α levels beyond what the tumor markers and stage already provided. 

T, N, and M categories did not individually retain significant effects (*p* > 0.10) on TNF-α once tumor markers were included, indicating that the relationship between advanced disease (especially T4 and M1) and TNF-α was largely captured by the elevated tumor marker levels. Similarly, AJCC stage (modeled as I–IV) was not an independent predictor of TNF-α (β = 0.45, 95% CI –0.52–1.42, *p* = 0.35) in the presence of the serological markers—reflecting the fact that much of the stage effect on TNF-α was encompassed by the elevated levels of CA19-9, CA72-4, and other factors, in advanced stages. 

**Figure 5:**
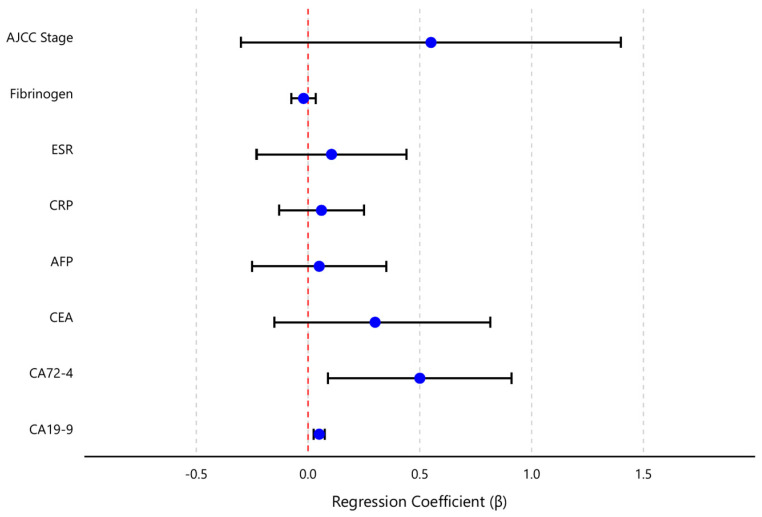
Forrest plot showing regression coefficients (β) within their 95% confidence intervals (CIs) for different predictors of TNF-α levels. Each row shows a predictor variable, with the blue dot representing the estimated β and the horizontal black line showing the CI. The vertical red dashed line at zero serves as a reference point—when a CI crosses this line, it suggests the predictor’s effect may not be statistically significant. The further a β is to the right of zero, the stronger its positive association with TNF-α levels; conversely, βs to the left of zero indicate negative associations. The width of the CIs indicates the precision of the estimates—narrower intervals suggest more precise estimates, while wider intervals indicate greater uncertainty.

Figure 5 provides a visual summary of the multivariate analysis, displaying the regression coefficients and their confidence intervals for each predictor. This coefficient plot highlights the dominant positive influence of CA19-9 and CA72-4 on TNF-α, in contrast to the near-zero effects of the other variables.

## 4. Discussion

Our study provides new insights into the interplay between an inflammatory cytokine (TNF-α) and established tumor markers in gastric cancer. In summary, we found that plasma TNF-α levels were significantly elevated in gastric cancer patients compared to controls (median 4.5 vs. 2.9 pg/mL, *p* = 0.014). More importantly, TNF-α showed strong positive correlations with several tumor markers within the cancer cohort. The most pronounced association was with CA19-9 (Spearman rho ≈ 0.50), followed by CA72-4 (rho ≈ 0.39) and CEA (rho ≈ 0.28), all reaching statistical significance. In contrast, no meaningful correlation was observed between TNF-α and AFP in our patients. These findings suggest that TNF-α elevation in gastric cancer is specifically linked to tumor burden indicators rather than to AFP, which is consistent with AFP’s limited role outside the rare AFP-producing gastric cancers [20,21]. Notably, none of these TNF-α–marker correlations were evident in the control group, underscoring that the TNF-α/tumor marker relationship is a malignancy-specific phenomenon and not a general feature of baseline physiology.

Our results align with and extend prior observations in the literature regarding tumor markers and cancer-associated inflammation. Elevated levels of CA19-9 and CEA are well-known indicators of advanced or aggressive gastric cancer. For instance, high preoperative CA19-9 has been shown to correlate with advanced disease stage, presence of metastases, and poorer survival [19]. Likewise, a meta-analysis by Deng et al. reported that elevated pretreatment CEA more than doubles the mortality risk in gastric cancer [17]. The robust TNF-α–CA19-9 correlation observed in our study reinforces the concept that TNF-α rises in tandem with tumor bulk and metastatic potential. In essence, TNF-α may be part of the same pathogenic milieu that leads to high CA19-9 in advanced disease. On the other hand, the lack of correlation between TNF-α and AFP is not surprising. AFP is typically elevated only in a distinct subset of gastric cancers (the AFP-producing variant) known for aggressive behavior. The majority of our cohort consisted of conventional gastric adenocarcinomas, in which AFP was seldom elevated; thus, the absence of a TNF-α–AFP relationship simply reflects that AFP was not a prevalent tumor marker in these patients. This underscores that TNF-α’s associations may be strongest with markers that are broadly relevant across gastric cancers (CEA, CA19-9, CA72-4) rather than those confined to special subtypes.

Importantly, we found that TNF-α levels differed according to tumor stage and histopathology, pointing to its potential role as a marker of disease severity. TNF-α exhibited a significant stage-dependent increase, with the highest levels observed in Stage IV disease (median ~8 pg/mL) compared to Stages I–III. In fact, TNF-α appeared to plateau in middle stages and then surge in Stage IV, suggesting that a substantial tumor burden or metastasis is required to provoke a marked systemic TNF-α response. This pattern is in agreement with other studies that have noted higher TNF-α expression in more advanced cancers [31]. Zhang et al., for example, recently demonstrated that gastric cancer patients with high preoperative TNF-α levels had significantly worse 5-year survival [26]. Taken together, these data support the notion that TNF-α is not only a bystander but may reflect the aggressiveness of the disease—rising as tumors invade deeply (T4) or spread to distant sites (M1). We also observed that CEA, among the tumor markers, increased significantly with stage in our cohort (particularly jumping in Stage IV), whereas CA19-9 and CA72-4 did not show clear stepwise increases. The parallel surge of TNF-α and CEA in Stage IV seemingly underscores their link to tumor progression. This could also imply that TNF-α and CEA are more consistently produced or released as the disease advances, whereas CA19-9 and CA72-4 might be influenced by additional factors or have high inter-patient variability in early stages. Overall, the stage-wise findings highlight TNF-α’s potential value in indicating an advanced disease state, complementing traditional staging and markers.

Notably, our observation that high TNF-α accompanies advanced disease aligns with studies in other cancers. For instance, colorectal cancer patients with elevated serum TNF-α had a dramatically shorter median survival (~7.8 months) compared to those with low TNF-α (~38.4 months) [32]. In breast cancer, high circulating TNF-α has likewise been identified as an independent predictor of poorer survival [33]. Such evidence across malignancies supports the concept that TNF-α reflects tumor aggressiveness and could be broadly prognostic.

Tumors of the intestinal subtype (Lauren classification) had the highest TNF-α concentrations, with a median level roughly double that of diffuse-type tumors. This subtype also showed the strongest TNF-α–CA19-9 correlation (rho ≈ 0.79) in a subset analysis, suggesting intestinal-type cancers might drive a particularly robust inflammatory (TNF-α) and tumor marker (CA19-9) response. One plausible explanation is that intestinal-type gastric cancers often arise on a background of chronic inflammation (e.g., long-standing H. pylori gastritis), which could prime the tumor microenvironment for higher TNF-α production. In contrast, diffuse-type and undifferentiated cancers (which are frequently associated with different molecular drivers, such as E-cadherin alterations) displayed lower systemic TNF-α levels [34]. Interestingly, in those subtypes, TNF-α correlated more with CEA (for diffuse) and very strongly with CEA in the undifferentiated type, indicating that even when overall TNF-α levels are lower, their relationship with certain markers persists. Similarly, when analyzing by WHO histopathological categories, we found that tubular adenocarcinomas (which largely correspond to Lauren intestinal-type) exhibited the highest TNF-α median values, whereas poorly cohesive carcinomas (related to the diffuse type) had more moderate TNF-α levels. Concordant with this, TNF-α in tubular adenocarcinoma cases was tightly linked to CA19-9, whereas in poorly cohesive carcinomas TNF-α was strongly correlated with CEA and CA72-4. These subtype-specific differences underscore that the relationship between cytokines and tumor markers can vary with tumor biology. From a clinical perspective, this means that a high TNF-α level might be especially indicative of aggressive disease in patients with intestinal-type tumors, whereas in diffuse-type tumors a modest TNF-α elevation could still be meaningful if accompanied by rises in CEA. This finding highlights the complex interactions between tumor biology and the host inflammatory response, warranting further investigation.

Our findings corroborate earlier work by our group and others while adding a new layer of understanding. In a previous study, Roșu et al. focused on TNF-α and CEA in gastric cancer and suggested that TNF-α has clinical significance in tandem with CEA levels [2]. We expand on that concept by demonstrating that TNF-α is also meaningfully linked with CA19-9 and CA72-4, which are critical markers for gastric cancer monitoring. Another recent analysis by Roșu et al. examined the importance of CA72-4 and CA19-9 in gastric cancer management [28]. Our current results tie these threads together, indicating that TNF-α elevations go hand-in-hand with increases in these key tumor markers. This integrated perspective is clinically relevant: measuring TNF-α alongside the conventional panel (CEA, CA19-9, CA72-4) could enhance diagnostic or prognostic accuracy. 

Indeed, it is known that combining multiple tumor markers improves detection and monitoring in gastric cancer. For example, simultaneous evaluation of CEA, CA19-9, and CA72-4 yields better diagnostic performance than any single marker alone [22]. The addition of TNF-α to such a panel might further stratify patients by aggressiveness of disease. However, it is important to note that traditional tumor markers might also participate in tumor progression. For example, CA72-4—like CA19-9 and CA125—may mediate tumor cell adhesion, aiding in metastasis [35]. Thus, an elevated CA72-4 level could worsen prognosis not just as a reflection of tumor burden, but by actively facilitating malignant spread. This concept could partly explain the strong correlation of TNF-α with CA72-4 and CA19-9 in our study: these markers and the cytokine might be interlinked in driving tumor aggressiveness.

Another interesting observation in our data was that TNF-α correlated preferentially with tumor-specific factors and showed only weak, non-significant correlations with systemic inflammation markers like CRP or ESR. This suggests that TNF-α in cancer patients is not merely a reflection of general inflammation or nutritional status, but is more tightly connected to tumor activity. In practical terms, TNF-α might serve as a tumor-focused biomarker—its elevation pointing more toward tumor-driven inflammation rather than, say, infection or benign inflammatory conditions (especially since we excluded patients with active inflammatory diseases in this study design).

From a biological standpoint, the prominent association of TNF-α with tumor markers and advanced disease in gastric cancer can be contextualized by TNF-α’s known roles in cancer biology. TNF-α is often described as a double-edged sword in oncology: while initially identified for its tumoricidal effects at very high concentrations, at lower levels such as those produced chronically in the tumor microenvironment, TNF-α tends to promote tumorigenesis [36]. It is a key mediator of cancer-related inflammation and has been implicated in several pro-tumoral processes. In gastric cancer, TNF-α can activate NF-κB and other pathways that lead to cancer cell proliferation, survival, and angiogenesis [13,37]. For example, in vitro experiments have shown that exposing gastric cancer cells to TNF-α enhances their invasive potential by downregulating protective factors like pentraxin-3 [37]. TNF-α also facilitates epithelial–mesenchymal transition and the recruitment of inflammatory cells that support metastasis [31,37]. Clinically, high TNF-α expression in tumor tissues or blood has been associated with adverse features such as lymph node involvement and early recurrence in various cancers [32]. Our observation that TNF-α is highest in metastatic (Stage IV) disease and correlates with markers like CA19-9 fits this paradigm: as the tumor burden increases, TNF-α levels rise, potentially aiding processes like tumor spread and immune evasion. Conversely, one must recall that TNF-α can induce apoptosis in cancer cells under certain conditions—however, those conditions (e.g., concentrated regional TNF-α administration) are not representative of the typical systemic TNF-α levels in patients [31,36]. Thus, in the context of untreated gastric cancer patients, the balance appears to tip towards TNF-α being a facilitator of tumor progression. This understanding lends biological plausibility to using TNF-α as a marker of aggressive disease: it is not only a correlate but likely an active participant in the cancer’s behavior.

Despite the strengths of our study (prospective design, simultaneous analysis of multiple biomarkers, and a well-defined patient cohort), several limitations should be acknowledged. First, the sample size, while powered for the primary correlation analysis, was modest (n = 80 cancer patients). This limited the statistical power for some subgroup analyses, such as comparisons across all histological subtypes or detailed stage subdivisions. For example, our Stage I subgroup had only 7 patients, which may explain why some trends did not reach significance. A larger multicenter cohort would be valuable to validate these findings and improve generalizability. Second, our study was cross-sectional—we measured TNF-α and tumor markers at a single time point (diagnosis) and did not track patient outcomes in this report. Therefore, we could not directly assess the prognostic value of TNF-α for survival or recurrence in our cohort. The strong associations with stage and known prognostic markers (CEA, CA19-9) suggest TNF-α could have prognostic significance, but longitudinal data are needed to confirm this hypothesis. Third, while we attempted to minimize confounding by excluding patients with active infections, autoimmune diseases, or other cancers, we did not specifically control for Helicobacter pylori infection status or other potential sources of chronic inflammation that might influence TNF-α. It is possible that some of the TNF-α elevation in certain patients was partially driven by such factors. Future studies could incorporate H. pylori status or measure additional cytokines (like IL-1β, IL-6) to dissect the contributions of tumor versus background inflammation. 

Another limitation is that our control group consisted of hospital patients with non-neoplastic conditions (rather than completely healthy individuals). We matched them for age, but because they were also undergoing surgery (e.g., for benign conditions), their baseline inflammatory marker levels might not have been entirely normal. However, the fact that TNF-α correlations were largely absent in controls argues that any minor inflammatory state in controls did not reproduce the patterns seen in cancer patients. Additionally, we were unable to evaluate the neutrophil-to-lymphocyte ratio (NLR) or absolute monocyte counts, as differential blood counts were not consistently available for our cohort. This precluded analysis of NLR, an inflammatory marker that has been linked to poor prognosis in gastric cancer [38]. The absence of NLR and monocyte data is a limitation of our study, as these could further illuminate the relationship between systemic inflammation and TNF-α levels. Lastly, on a technical note, TNF-α was measured by ELISA in our study. While this is a standard and sensitive method, measurements of cytokines at low concentrations can be subject to variability. We mitigated this by strict sample handling protocols, but replication using high-sensitivity assays or multiplex cytokine panels would strengthen confidence in the exact values and cutoffs reported. 

Looking ahead, our findings pave the way for several future research directions. One important next step is to evaluate the prognostic utility of TNF-α in a longitudinal manner. It would be valuable to follow gastric cancer patients over time to see if baseline or postoperative TNF-α levels predict outcomes like recurrence-free survival or overall survival. Given the link between high TNF-α and advanced disease, we hypothesize that patients with elevated TNF-α might experience earlier recurrences or metastases; this should be tested in a dedicated follow-up study. Additionally, integrating TNF-α into prognostic models alongside tumor markers and inflammatory indices could improve risk stratification. For example, a composite score combining TNF-α, CA19-9, and perhaps IL-6 (another cytokine tied to gastric cancer prognosis [26]) might better identify high-risk patients than any single marker. Another avenue is exploring TNF-α as a monitoring biomarker. Serial TNF-α measurements during treatment could inform whether a patient’s inflammatory tumor milieu is responding—for instance, a drop in TNF-α after surgery or chemotherapy might correlate with tumor reduction, whereas persistently high or rising TNF-α could signal occult disease. This approach would require carefully designed prospective trials.

Finally, our study raises considerations for therapeutic interventions targeting TNF-α in gastric cancer. Interestingly, several existing drugs have been shown to lower TNF-α levels. For example, bupropion, a commonly used antidepressant, significantly suppresses TNF-α production in vivo [39]. Similarly, dapsone (an anti-inflammatory antibiotic) can downregulate TNF-α release from activated immune cells by ~54% [40]. The phosphodiesterase-4 inhibitor roflumilast, approved for COPD, also reduces LPS-induced TNF-α secretion in human tissues [41]. Moreover, pomalidomide—an immunomodulatory drug—is a potent TNF-α inhibitor (up to 50,000-fold more potent than thalidomide) [42]. These examples highlight the feasibility of pharmacologically targeting TNF-α, supporting further exploration of anti-TNF strategies in gastric cancer.

The concept of anti-TNF therapy (using agents like infliximab or etanercept) has shown promise in other malignancies and inflammatory conditions by reducing tumor-promoting inflammation [43,44,45]. While such agents have not yet been applied in routine gastric cancer treatment, it would be intriguing to investigate them in a research setting. Patients with metastatic gastric cancer and high TNF-α levels could potentially be candidates for trials adding an anti-TNF drug to standard chemotherapy or immunotherapy. The rationale is supported by our data and the broader understanding that TNF-α fosters a pro-tumor microenvironment; neutralizing it might impair the tumor’s support network. Of course, safety and efficacy would need thorough evaluation—prior trials in other cancers have had mixed results, and TNF-α blockade can carry risks (e.g., infection). Nonetheless, as immunotherapy becomes more prominent in gastric cancer, there may be synergy in concurrently modulating the tumor’s cytokine milieu. In summary, further exploration of TNF-α—from its prognostic value to its potential as a treatment target—is warranted to determine how this multifaceted cytokine can be leveraged to improve gastric cancer outcomes.

## 5. Conclusions

In conclusion, our study demonstrates that TNF-α levels are significantly elevated in gastric cancer patients and closely correlate with key tumor markers (especially CA19-9 and CA72-4), reflecting the tumor burden and aggressiveness of the disease. TNF-α also varied with histopathological subtype and disease stage, being highest in intestinal-type and advanced-stage cancers. These findings underscore the clinical significance of TNF-α as a potential biomarker in gastric cancer. TNF-α could serve as a useful adjunct to traditional tumor markers, aiding in patient stratification and risk assessment. For instance, a high TNF-α level at diagnosis may flag patients with a more advanced inflammatory tumor milieu who might benefit from intensive monitoring or therapy, whereas low TNF-α could suggest a more indolent course. Incorporating TNF-α measurement into clinical practice—for example, as part of a preoperative work-up or a post-treatment surveillance panel—could enhance the detection of residual disease or impending relapse when used alongside CEA, CA19-9, and CA72-4.

Moreover, our results provide a rationale for exploring therapeutic interventions targeting TNF-α in gastric cancer. Given its association with tumor progression, TNF-α represents a novel target for anti-cancer therapy; strategies to inhibit TNF-α (or its downstream effects) might suppress the pro-tumor inflammation that fuels disease spread. In the future, patients with elevated TNF-α might be considered for clinical trials evaluating anti-TNF agents as an adjunct to standard treatment. TNF-α is an integral component of the gastric cancer microenvironment, both reflecting and potentially driving disease severity. Recognizing the role of TNF-α may help clinicians better stratify patients and develop more personalized, targeted management strategies for gastric cancer. Further studies, especially longitudinal cohorts and interventional trials, are warranted to validate TNF-α as a prognostic biomarker and to determine whether modulating its activity can improve patient outcomes.

## Figures and Tables

**Table 1:** Comparison of patient characteristics and laboratory values between groups.

	Gastric Cancer (n = 80)	Control Group (n = 20)	*p*-Value
Age, years *	68.0 [60.0–76.0]	66.5 [61.25–68.75]	0.45
Male sex, n (%)	49 (61.3)	10 (50.0)	0.921
Hemoglobin, g/dL *	10.5 [9.2–12.2]	13.9 [13.2–14.9]	<0.001
WBC, ×10^3^/μL *	8.2 [6.3–10.2]	7.2 [5.8–9.1]	0.137
Total protein, g/dL *	6.1 [5.4–6.7]	6.9 [6.6–7.4]	<0.001
Albumin, g/dL *	3.4 [2.9–3.9]	4.2 [3.8–4.5]	<0.001
Creatinine, mg/dL *	0.8 [0.7–1.0]	0.9 [0.7–1.0]	0.521
ALT, U/L *	12.6 [8.7–18.3]	15.0 [12.1–29.0]	0.051
AST, U/L *	17.3 [13.2–24.8]	19.5 [14.8–26.2]	0.639
GGT, U/L *	24.0 [15.0–39.0]	19.0 [13.0–39.0]	0.806
ALP, U/L *	73.0 [63.0–85.0]	62.0 [53.0–90.0]	0.092
CRP, mg/L *	16.2 [5.6–44.0]	1.6 [1.2–4.2]	<0.001
ESR, mm/h *	26.0 [18.0–42.0]	15.0 [10.0–25.0]	0.001
Fibrinogen, mg/dL *	419.0 [350.0–509.0]	329.0 [286.0–398.0]	0.003
PCT, ng/mL *	0.06 [0.04–0.10]	0.03 [0.02–0.04]	<0.001
TNF-α, pg/mL *	4.5 [2.8–11.4]	2.9 [1.7–5.7]	0.014
CEA, ng/mL *	2.5 [1.5–5.5]	2.0 [1.3–2.9]	0.077
CA19-9, U/mL *	12.1 [6.1–25.1]	6.4 [3.2–10.6]	0.030
CA72-4, U/mL *	4.9 [3.1–7.9]	2.4 [1.8–4.2]	<0.001
AFP, ng/mL *	1.7 [1.2–3.1]	1.5 [1.0–2.4]	0.126

* Values presented as median [interquartile range]. Abbreviations: WBC = white blood cell count; ALT = alanine aminotransferase; AST = aspartate aminotransferase; GGT = gamma-glutamyl transferase; ALP = alkaline phosphatase; CRP = C-reactive protein; ESR = erythrocyte sedimentation rate; PCT = procalcitonin; TNF-α = tumor necrosis factor alpha; CEA = carcinoembryonic antigen; CA = cancer antigen; AFP = alpha-fetoprotein. Note: *p*-values were calculated using Mann–Whitney U test for continuous variables and chi-square test for categorical variables.

**Table 2:** Comparative TNF-α correlation analysis between study and control groups.

	Gastric Cancer (n = 80)		Control Group (n = 20)	
Correlation with TNF-α	Rho	*p*-Value	Rho	*p*-Value
CEA (ng/mL)	0.279	0.0122 *	−0.020	0.9348
CA72-4 (U/mL)	0.385	0.0004 **	0.150	0.5280
CA19-9 (U/mL)	0.502	<0.0001 **	0.370	0.1084
AFP (ng/mL)	0.070	0.5358	0.383	0.0956
CRP (mg/L)	0.204	0.0698	−0.226	0.3371
Fibrinogen (mg/dl)	0.031	0.7847	−0.426	0.0614
Hemoglobin (g%)	0.058	0.6072	0.066	0.7814
Total Proteins (g/dL)	0.040	0.7265	0.161	0.4978
TNF-α (pg/mL)	1	-	1	-

** Correlation is significant at the 0.01 level (2-tailed); * correlation is significant at the 0.05 level (2-tailed). Spearman’s rank correlations. Abbreviations: TNF-α = tumor necrosis factor alpha; CEA = carcinoembryonic antigen; CA = cancer antigen; AFP = alpha-fetoprotein; CRP = C-reactive protein.

**Table 3:** Stratification of TNF-α, tumor markers, and inflammatory parameters by AJCC TNM stage.

Marker	Stage I (n = 7)	Stage II (n = 15)	Stage III (n = 24)	Stage IV (n = 34)	*p*-Value (K-W)
TNF-α ^+^	5.7 [2.84–9.90]	4.2 [2.61–9.80]	4.1 [2.10–7.80]	8.0 [3.25–14.60]	0.037 *
CA72-4 ^+^	5.4 [5.04–7.15]	5.2 [2.10–7.90]	4.1 [2.30–7.30]	4.8 [3.10–8.40]	0.330
CEA ^+^	2.8 [1.04–5.17]	2.3 [1.71–5.44]	1.7 [1.15–3.41]	3.7 [1.63–21.93]	0.007 *
CA19-9 ^+^	12.1 [5.20–334.81]	13.6 [6.96–25.61]	10.8 [2.72–17.20]	13.0 [6.17–25.00]	0.084
AFP ^+^	2.4 [1.70–3.40]	1.8 [1.20–2.60]	1.5 [1.20–2.37]	2.0 [1.21–3.20]	0.195
CRP ^+^	2.4 [0.57–18.40]	26.0 [5.60–41.02]	10.2 [2.96–40.00]	28.3 [8.39–64.63]	0.044 *
ESR ^+^	24.0 [20.00–30.00]	30.0 [18.00–50.00]	24.00 [16.00–50.00]	30.00 [22.00–42.00]	0.308
Fibrinogen ^+^	395 [373–501]	470 [342–501]	405 [349–595]	419 [352–524]	0.877

^+^ Values presented as median [interquartile range]. Abbreviations: TNF-α = tumor necrosis factor alpha; CEA = carcinoembryonic antigen; CA = cancer antigen; AFP = alpha-fetoprotein; K-W = Kruskal–Wallis test. Note: *p*-values were calculated using the Kruskal–Wallis test. * Statistically significant after Benjamini–Hochberg correction for multiple comparisons (false discovery rate < 0.05).

**Table 4:** Multiple linear regression analysis predicting TNF-α levels (pg/mL) from tumor markers, inflammatory markers, and AJCC stage in gastric cancer patients.

Predictor	β Coefficient (pg/mL Per Unit Increase)	95% CI	*p*-Value
**CA19-9 (U/mL)**	**0.10**	**0.05 to 0.15**	<0.001 *
**CA72-4 (U/mL)**	**0.50**	**0.18 to 0.82**	0.003 *
CEA (ng/mL)	0.30	–0.03 to 0.63	0.075
AFP (IU/mL)	0.01	–0.05 to 0.07	0.702
CRP (mg/L)	0.012	–0.026 to 0.050	0.53
ESR (mm/h)	0.021	–0.046 to 0.088	0.53
Fibrinogen (mg/dL)	–0.004	–0.015 to 0.007	0.45
AJCC Stage (I–IV, ordinal)	0.45	–0.52 to 1.42	0.35

* *p* < 0.01. Significant predictors (*p* < 0.05) are in bold. Shown are the estimated regression coefficients (β) for each predictor, with 95% confidence intervals and *p*-values. Model R^2^ = 0.36 (adjusted R^2^ = 0.30), *p* < 0.001. The coefficients represent the change in TNF-α (pg/mL) per one-unit increase in each predictor (for AJCC stage, per increment in stage class). Only CA19-9 and CA72-4 showed significant independent associations with TNF-α after controlling for all other variables.

## Data Availability

Data available on request.

## Abbreviations

The following abbreviations are used in this manuscript:

AFP	Alpha-fetoprotein
AJCC	American Joint Committee on Cancer
ALP	Alkaline phosphatase
ALT	Alanine aminotransferase
AST	Aspartate aminotransferase
CA19-9	Cancer antigen 19-9
CA72-4	Cancer antigen 72-4
CEA	Carcinoembryonic antigen
CI	Confidence interval
CRP	C-reactive protein
CT	Computed tomography
ECLIA	Electrochemiluminescent immunoassay
EGD	Esophagogastroduodenoscopy
ELISA	Enzyme-linked immunosorbent assay
ESMO	European Society for Medical Oncology
ESR	Erythrocyte sedimentation rate
GGT	Gamma-glutamyl transferase
IQR	Interquartile range
MRI	Magnetic resonance imaging
NK	Natural killer
NOS	Not otherwise specified
pTNM	Pathologic TNM
TNF-α	Tumor necrosis factor alpha
WHO	World Health Organization
